# Sciatica in Early Pregnancy With Coexisting Uterine Leiomyoma and‌ ‌Tarlov‌ ‌Cyst:‌ ‌A‌ ‌Case Report

**DOI:** 10.7759/cureus.27855

**Published:** 2022-08-10

**Authors:** Efe S Disi

**Affiliations:** 1 College of Medicine, Madonna University, Elele, NGA; 2 Biology, University of Kentucky, Lexington, USA

**Keywords:** uterine leiomyoma, radiculopathy in pregnancy, laparoscopic surgery in pregnancy, tarlov cyst, sciatica, fibroid, first trimester pregnancy

## Abstract

Uterine fibroids are common, especially among women of African descent. Several women with fibroids are asymptomatic, which may contribute to underestimating its prevalence. Symptomatic uterine fibroids present with menstrual problems, anemia, infertility, miscarriages, an enlarged abdomen, pressure symptoms involving the bladder and bowels (such as frequent urination or constipation), and sometimes coital-related problems. This case report describes a 25-year-old African American woman with uterine fibroids who suffered from lower back pain radiating to the left lower extremity, along with paresthesias and weakness of the left leg. She was diagnosed with lumbar radiculopathy, early pregnancy, and an incidental finding of a Tarlov cyst. As the pregnancy progressed, the sciatic pain ceased within the first trimester. Sciatic pain can result from a sudden increase in the uterus size caused by an early pregnancy coexisting with large fibroids. The sciatic pain may not remain throughout the pregnancy as the growing uterus with large fibroids may be displaced from the site of nerve compression.

## Introduction

Uterine leiomyoma, also known as uterine fibroid, is a benign neoplasm composed of mostly smooth muscle mass that usually develops during a woman's reproductive years. Of all the symptoms caused by uterine fibroids, bleeding symptoms are the most common. Pressure symptoms are among the least common and are usually related to the bladder or rectum rather than surrounding nerves. There are a few case reports on post-menopausal women with uterine fibroids causing lumbosacral plexopathy, gait disorder, and lumbar radiculopathy, in which a hysterectomy relieved their symptoms [1-3]. This case report presents a 25-year-old African American woman with sciatica triggered by an enlarging pregnant uterus coexisting with fibroid, which was later relieved as the pregnancy progressed in size and age.

## Case presentation

A 25-year-old nulliparous African American female with a medical history of uterine fibroids and benign microscopic hematuria presented with gradual onset lower back pain radiating to her left lower extremity. This pain progressively worsened, associated with paresthesias of the left lower extremity, and was not improved by non-steroidal anti-inflammatory drugs. The patient was sexually active and not on contraception. The patient had a regular menstrual period.

**Figure 1:**
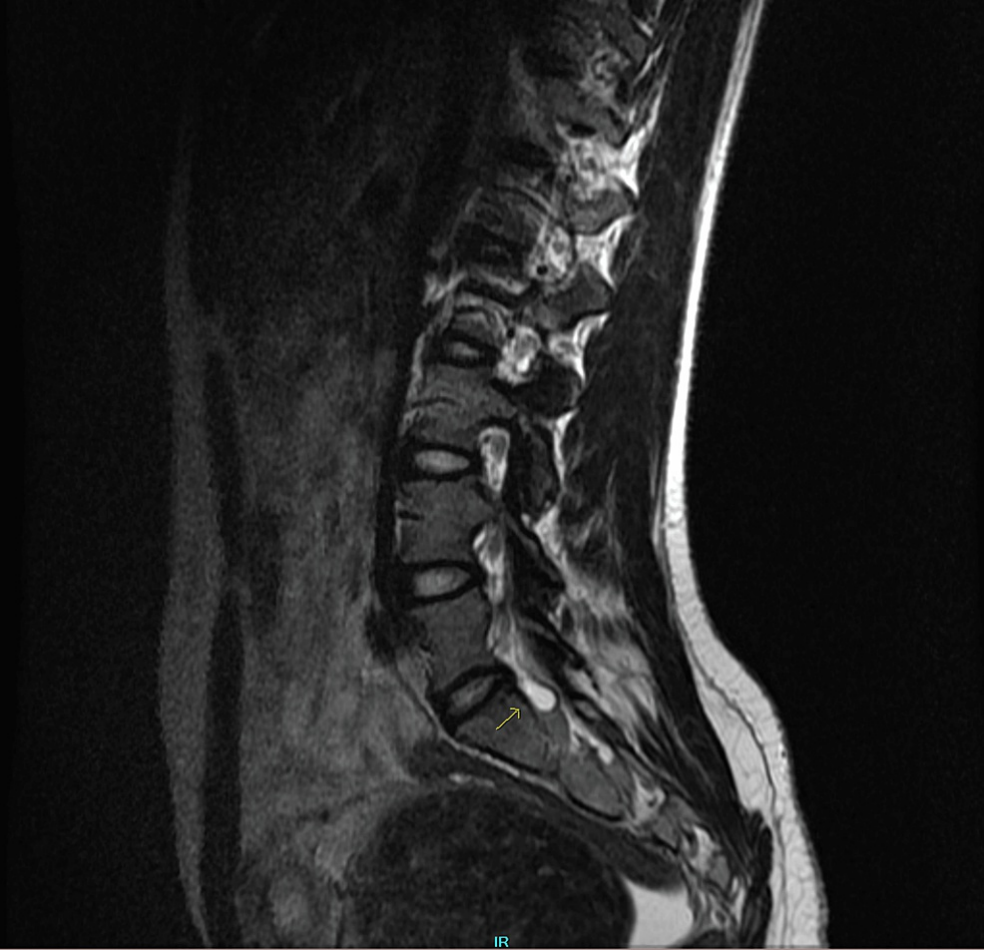
Sagittal lumbar MRI showing a tiny Tarlov cyst indicated by an arrow.

**Figure 2:**
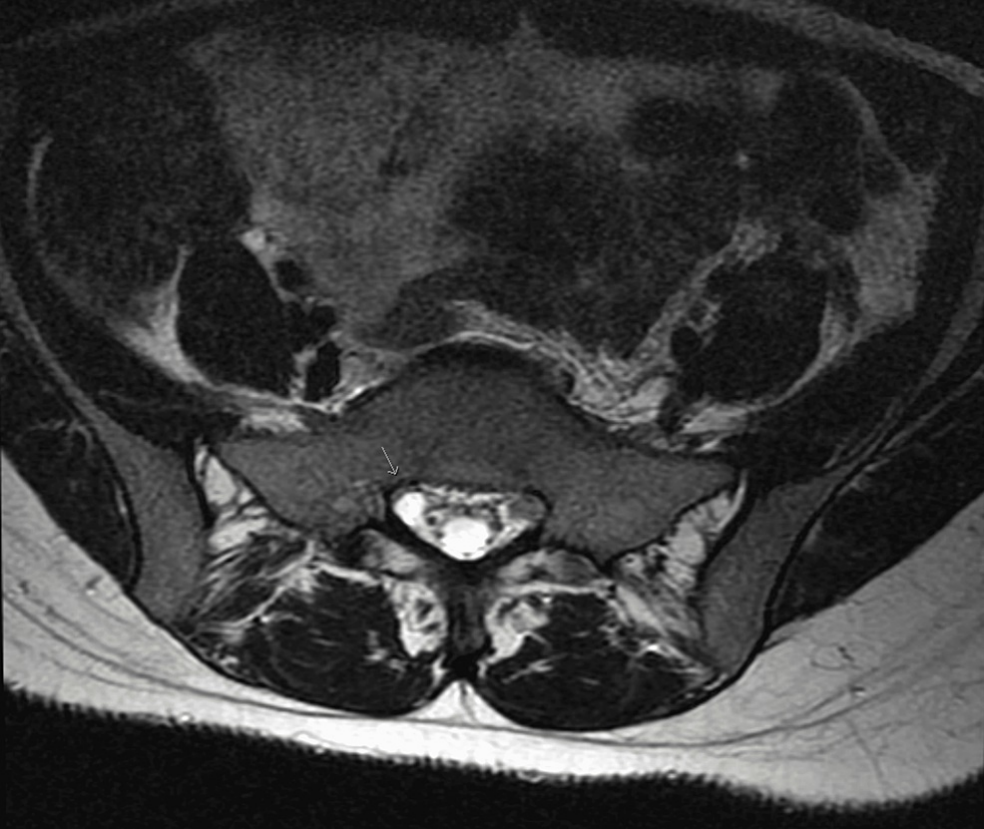
Axial lumbar MRI showing a tiny Tarlov cyst on the right side indicated by an arrow.

**Figure 3:**
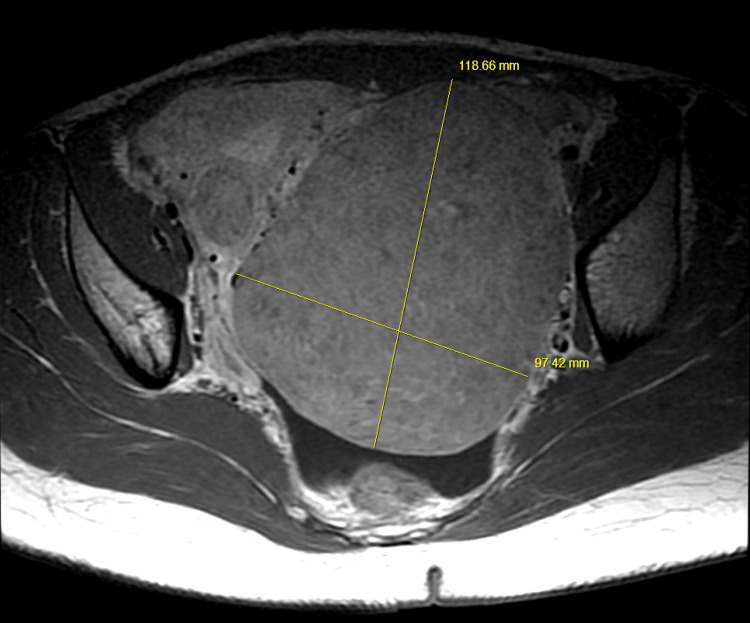
Axial pelvic MRI showing a large uterine fibroid measuring approximately 120 x 100 mm. Structures (sciatic nerve) in between the fibroid and piriformis muscle on the left side appear compressed compared to the right side.

**Figure 4:**
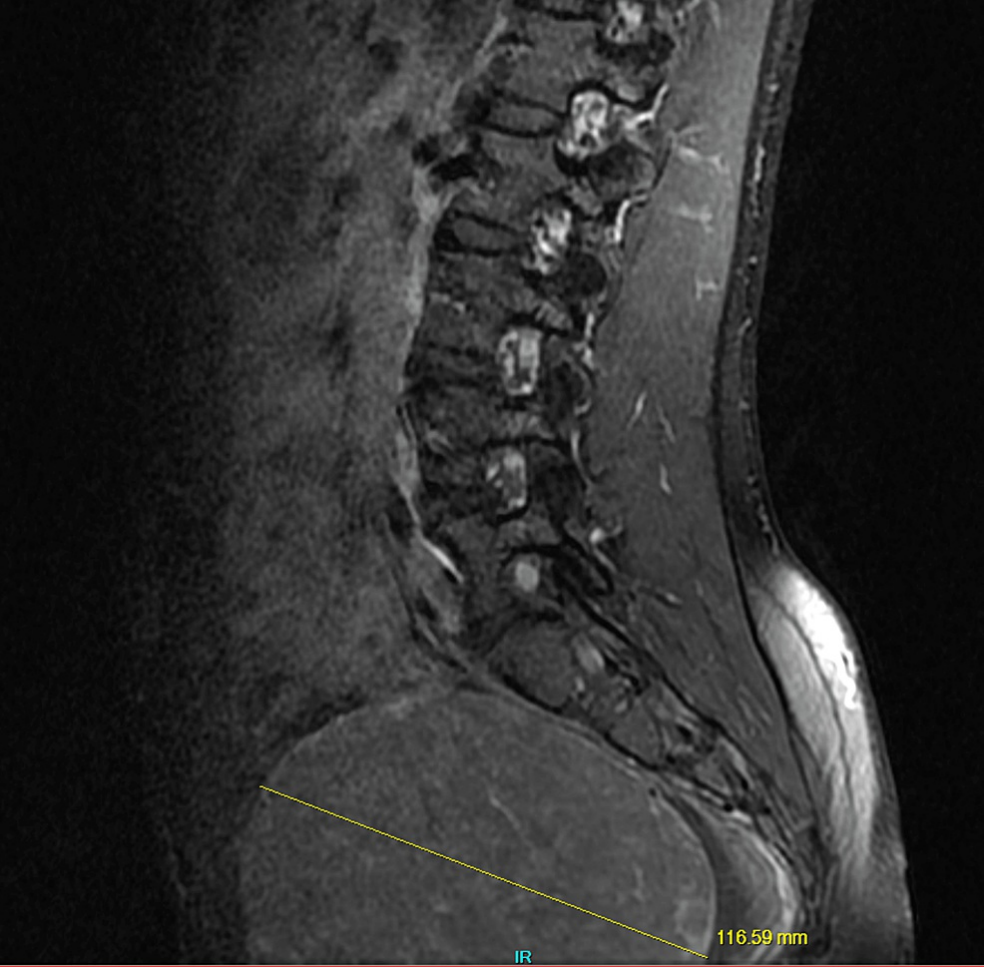
Sagittal lumbar MRI showing a large uterine fibroid in the pelvic region measuring approximately 120 mm.

On physical examination, the left lower lumbar and gluteal regions were tender, the range of motion on the left hip was abnormal, and a straight leg test was positive on the left (flexion of hip with knees in extension elicited pain). Muscle strength, sensation, and reflexes were normal. The patient was diagnosed with radiculopathy, placed on tramadol 50 mg to be taken orally three times daily, and scheduled for a magnetic resonance imaging (MRI) of the lumbar spine with and without contrast. The MRI showed no disc protrusion or stenosis, although a tiny Tarlov cyst was present at L5-S1 on the right (Figures 1, 2). The MRI scan also showed a large soft tissue pelvic mass measuring approximately 12 x 10 x 12 cm, very likely consistent with her history of fibroid (Figures 3, 4).

Five days later, a follow-up with the patient showed no pain relief with tramadol, and the patient complained of worsening symptoms that included weakness of the left lower extremity. She also stated her concern about a missed menstrual period of three days. There was a positive urine pregnancy test. The patient appeared thin with a slightly prominent lower abdomen on physical examination. On palpation, there was tenderness in the left lower pelvic and the left hip regions. There was also decreased range of motion of the left hip during flexion. An abdominal and vaginal ultrasound showed no gestational sac in the uterus. Gestational age was four weeks and three days based on her last menstrual period, and beta human chorionic gonadotropin (hCG) was 4,800 IU/L. Given the following results, the patient was diagnosed with an abnormal pregnancy (suspected ectopic pregnancy) and scheduled for diagnostic laparoscopy.

Intraoperatively, there was a large, approximately 10-cm fibroid extending from the entire posterior surface of the uterus to the patient's left pelvic side wall. Pregnancy could not be identified anywhere in the pelvis. The patient recovered well from surgery; however, she still had sciatica and was placed on oxycodone, improving some symptoms. It is important to note that the patient had an early intrauterine pregnancy, which was challenging to visualize, most likely due to the technical difficulties of ultrasound with the fibroids. Subsequent follow-up with an abdominal ultrasound revealed intrauterine pregnancy at five weeks five days gestation. Around the seventh week of pregnancy, the patient's sciatic pain resolved without further pain medication.

## Discussion

Sciatica is not an uncommon complaint during pregnancy. During pregnancy, the hormone relaxin relaxes the ligaments causing a shift in the body's center of gravity, which can lead to irritation or a pinch in the sciatic nerve [4]. In addition to the fibroids, the relaxin hormone may have played a role in the patient's sciatica because its levels are highest in the first trimester of pregnancy. Although sciatica can happen in any trimester, it is more likely during the third trimester as the baby's weight might add pressure to the nerve [4].

The incidental finding of a Tarlov cyst on the patient's MRI scan could have been a possible cause of the sciatic pain. Tarlov cysts are fluid-filled nerve root sacs usually asymptomatic and incidentally found on MRI scans. In some cases, the cysts could expand and put pressure on affected nerve roots presenting with sciatic pain, weakness, and decreased sensation along the affected lower extremity. Spontaneous resolution of Tarlov cyst and sciatic pain can occur, which is a possible explanation for why her symptoms were resolved without surgical intervention [5]. However, the MRI showed that the Tarlov cyst was tiny and was located on the right side (Figure 2), while her symptoms were on the left side. As a result of its size and location, it is an improbable cause of the sciatic symptoms she was experiencing on the left.

The large uterine fibroid seemed more likely based on its location, as indicated ‌‌on ‌the‌ MRI scan and ‌intraoperatively. ‌It extended from the posterior wall of the uterus to the left pelvic wall, corresponding to the point of nerve compression on the left (Figure 3). Cases of large fibroids causing radiculopathy and gait disorder have been resolved by hysterectomy, although these cases were in perimenopausal and post-menopausal women [1-3]. During menopause, uterine fibroids tend to regress, alleviating symptoms, but sometimes fibroids occur within this age group, especially among African Americans [6]. In a cohort study by Templeman et al., the second incidence of uterine fibroid was among women aged 50-54 years [7]. However, in the case of this 25-year-old, her symptoms subsided in the first trimester as the pregnancy grew in size. During pregnancy, the uterus grows up and out of the pelvis around 12 weeks gestation [8]. Keep in mind that the uterine size did not correspond to the expected size at each gestational age because of the coexisting fibroids. The patient's uterus was already palpable before 12 weeks gestation. In addition, uterine fibroids tend to increase in size during pregnancy because of increased maternal sex hormones, and they grow fastest before 11-14 weeks of gestation [9]. It is, therefore, possible that the accelerated growth of the fibroid mass during the first trimester with the growing uterus impacted pressure on the sciatic nerve, and fibroid mass was displaced as the uterus grew out of the pelvis, thereby relieving pressure on the nerve.

Complications in pregnancies co-existing with fibroids usually depend on the size and location of the fibroid. Some adverse obstetric outcomes, in order of prevalence, include cesarean section, malpresentation, labor dystocia, postpartum hemorrhage, peripartum hysterectomy, and preterm labor and delivery [10]. Sciatica is an unusual symptom of fibroid, especially in pregnancy. Although the pain resolved during the first trimester, it would not be unusual to anticipate a recurrence during the pregnancy or even puerperium as the uterus shrinks back to a near pre-pregnancy state, being around the same size at the onset of the sciatic pain. In this patient's case, there was no recurrence during and after pregnancy; however, she experienced red degeneration of the fibroid and bleeding in the second trimester. She went into preterm labor and was delivered at 35 weeks gestation vaginally. A reoccurrence of sciatica caused by fibroid in pregnancy is not improbable as it would depend on some factors. The probability of sciatica recurring may depend on the uterus's change in position during pregnancy and puerperium. Furthermore, the possibility of a reduction in the size of the fibroid during puerperium could be another factor. Most fibroids show no change during the puerperium; however, about 7.8% will reduce in volume by up to 10% [11,12]. A reduced fibroid volume and a change in the uterus's position could have reduced the chances of this patient's sciatica reoccurring prenatal or postpartum.

## Conclusions

A focused obstetrical and gynecological history should be considered in a female patient presenting with sciatic pain, and further examination and investigation should be conducted based on historical findings. In managing a patient with early pregnancy and a history of fibroids presenting with sciatic pain, one should consider conservative management such as analgesic control and close monitoring after ruling out other probable causes.
